# Endosperm Tolerance of Paternal Aneuploidy Allows Radiation Hybrid Mapping of the Wheat D-Genome and a Measure of γ Ray-Induced Chromosome Breaks

**DOI:** 10.1371/journal.pone.0048815

**Published:** 2012-11-07

**Authors:** Vijay K. Tiwari, Oscar Riera-Lizarazu, Hilary L. Gunn, KaSandra Lopez, M. Javed Iqbal, Shahryar F. Kianian, Jeffrey M. Leonard

**Affiliations:** 1 Department of Crop and Soil Science, Oregon State University, Corvallis, Oregon, United States of America; 2 International Crops Research Institute for the Semi-Arid Tropics (ICRISAT), Patancheru, Andhra Pradesh, India; 3 Department of Plant Sciences, North Dakota State University, Fargo, North Dakota, United States of America; University of New England, Australia

## Abstract

Physical mapping and genome sequencing are underway for the ≈17 Gb wheat genome. Physical mapping methods independent of meiotic recombination, such as radiation hybrid (RH) mapping, will aid precise anchoring of BAC contigs in the large regions of suppressed recombination in Triticeae genomes. Reports of endosperm development following pollination with irradiated pollen at dosages that cause embryo abortion prompted us to investigate endosperm as a potential source of RH mapping germplasm. Here, we report a novel approach to construct RH based physical maps of all seven D-genome chromosomes of the hexaploid wheat ‘Chinese Spring’, simultaneously. An 81-member subset of endosperm samples derived from 20-Gy irradiated pollen was genotyped for deletions, and 737 markers were mapped on seven D-genome chromosomes. Analysis of well-defined regions of six chromosomes suggested a map resolution of ∼830 kb could be achieved; this estimate was validated with assays of markers from a sequenced contig. We estimate that the panel contains ∼6,000 deletion bins for D-genome chromosomes and will require ∼18,000 markers for high resolution mapping. Map-based deletion estimates revealed a majority of 1–20 Mb interstitial deletions suggesting mutagenic repair of double-strand breaks in pollen provides a useful resource for RH mapping and map based cloning studies.

## Introduction

Direct sequencing efforts of the wheat (*Tritcum aestivum* L.) genome are complicated by its large size (∼17 Gb), hexaploid nature, and high repeat content (∼80%). Various methods have been employed to assemble BAC contigs prior to BAC-by-BAC sequencing [1]. More recently, high-throughput sequencing has encouraged whole genome sequencing (WGS) approaches [2], although assembly of hundreds of millions of short sequence into large contigs is computationally difficult. Regardless of the approach, thousands of gaps in the final sequence will require a robust physical map to precisely assemble contigs in an accurate order [3].

Radiation hybrid (RH) mapping was developed to map the human genome [4], [5] and has become a well-established technique utilized extensively as an alternative to genetic mapping [6], [7], [8], [9]. It is based on radiation-induced chromosome breakage rather than meiotic recombination as a means to induce marker segregation for mapping [4], [10], [11]. A panel of independently derived radiation hybrids is assayed for the presence or absence of markers, and the patterns and frequencies of marker co-retention are used to calculate their physical proximity to establish a physical map [11]. Assuming random radiation-induced breakage, RH map distances (centiRays) more accurately reflect the physical distance between markers than genetic maps [12], [13]. Initially, RH maps of single chromosomes were constructed and therefore required multiple panels to map all chromosomes of a given species. To overcome these limitations, a variant of the RH mapping approach, whole-genome radiation hybrid mapping (WGRH), was developed to allow analysis of entire genomes [14], [15].

In plants, the use of RH mapping has been limited [16]. Riera-Lizarazu et al [17] first applied RH mapping in plants using oat-maize addition lines. Wardrop et al. [18] reported a method based on somatic cell fusion for construction of a whole genome radiation hybrid panel for barley; however, the utility of these hybrids is limited by technical difficulties in their generation and continuous culture [19], [20]. RH mapping based on seed irradiation was applied to build a physical map of wheat chromosome 1D [16], [21]. However this work was limited to a single chromosome. Riera-Lizarazu et al. [22] reported an approach based on seed irradiation to construct RH maps of all seven wheat D-genome chromosomes simultaneously. However, retention frequencies reported in the seed-irradiation panels are quite high (∼97%) for any given marker. The low frequency (∼5%) of low-marker-retention lines required for RH mapping produced by seed irradiation suggested the need for an alternate approach of RH production.

Pollen irradiation is a widely used technique in crop species to induce mutations and gene transfer [23], [24], [25], [26]. It disturbs double fertilization, and subsequently the development and interaction of embryo and endosperm, in a dose-related manner [27]. Because radiation-induced chromosome damage occurs post meiosis, selection against chromosome aberrations both during plant growth and gametogenesis is minimized allowing maximum recovery of deletions. In seeds, most radiation dosage studies have focused on the effect of radiation on embryo development while largely ignoring endosperm development [28], [29]. However, in *Actinidia deliciosa,* the proportion of seeds containing only endosperm, produced following pollen irradiation (900 Gy), was almost ten-fold higher (22.8%) than those containing both (2.8%) embryo and endosperm [30]. The independent response of embryo and endosperm development and apparent high tolerance of endosperm for paternal chromosomal aberrations suggested an alternate route of RH mapping panel development in plants.

We followed the scheme developed by Riera-Lizarazu et al [22] to isolate D-genome chromosomes in a monosomic condition by fertilizing the tetraploid wheat Altar 84 with irradiated pollen from the hexaploid wheat Chinese Spring. The development of both endosperm and embryo were measured after fertilization with pollen treated with varying dosages of γ rays. As suggested by studies in *Actinidia*, pollen treated with a radiation dose that yielded no viable embryos, still produced developed endosperm. To test the potential utility for RH mapping, a subset of endosperm samples was genotyped with DArT markers, [31], [32], [33], [34], and that data was used to generate physical maps for all seven D-genome chromosomes of wheat. This method offers a quickly developed resource for the dense physical maps required for alignment of contigs prior to sequencing the wheat genome. The RH maps will also offer opportunities to study DSB repair in pollen with large data sets.

## Results

### Endosperms Tolerate More Paternal Radiation-induced Chromosome Aberrations than Embryos

Seed set on tetraploid spikes pollinated with non-irradiated Chinese Spring pollen was 80% but decreased significantly (*p*<0.01) with increased radiation dosages. D-genome Radiation Hybrid F_1_ (DGRH_1_) seed set for 10-, 15-, 20-, 25- and 30-Gy pollen-irradiation treatments was 59.2, 35.3, 21.1, 2.0 and 0%, respectively. Successful seed set always included endosperm development. Endosperm development was higher than embryo development in all radiation treatments. Successful embryo development fell from 80% at 0-Gy, to 46.8, 13.4 and 0% at 10-, 15-, and 20-Gy treatments respectively.

DNA was extracted directly from endosperm derived from 10-, 15-, and 20-Gy irradiated pollen (endosperm panels). We grew and extracted DNA from 282 DGRH_1_ plants derived from 15-Gy irradiated pollen (plant panel). Estimations of radiation-induced deletions were made by measuring retention rates of 14 D-genome specific SSRs on both endosperm and plant panels. Average marker retention rates in endosperm panels decreased with increased radiation dosages. Significantly different average marker retentions of 92.6%, 84.1% and 68.0% were measured for endosperm panels from a 48-member 10-Gy panel, a 51-member 15-Gy panel, and a 282-member 20-Gy panel, respectively (*p*<0.01 between 10-Gy and 15-Gy and *p<*0.05 between 15-Gy and 20-Gy). Average marker retention of the 15-Gy plant panel was 86.2%.

### Radiation Hybrid Based Physical Maps can be made with Endosperm

**Figure 1:**
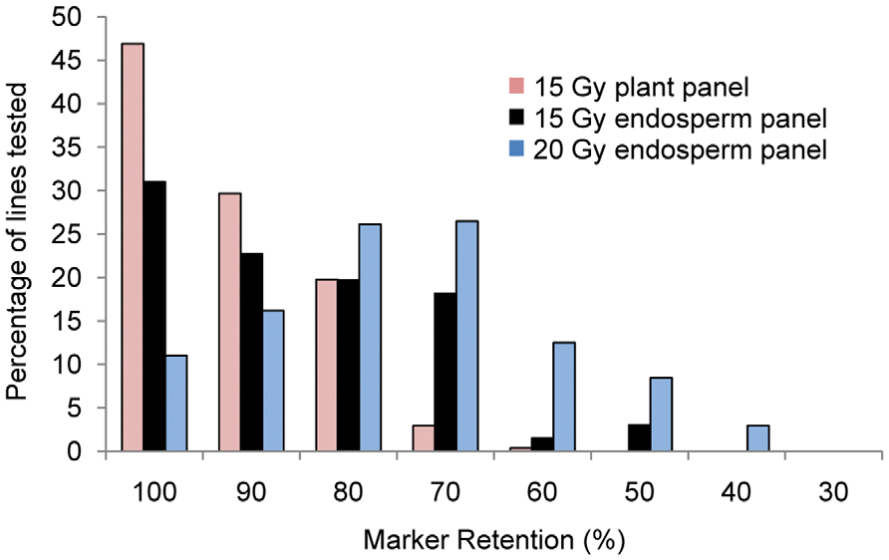
Distribution of marker retention rates following pollen irradiation. A panel of 14 SSR markers was used to estimate radiation-induced deletion rates from plants derived from 15-Gy irradiated pollen and from endosperm derived form 15- or 20-Gy irradiated pollen. Marker retention was calculated as the ratio of missing markers to total number of markers successfully assayed for each line. Embryos failed to develop following pollination with 20-Gy treated pollen and were not included in this study.

**Figure 2:**
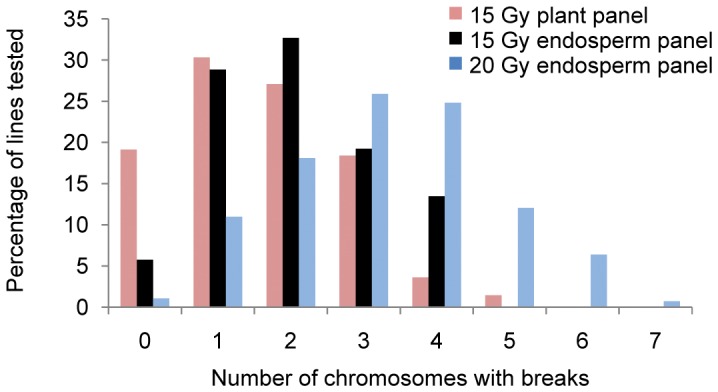
Distribution of RH lines with deletions in multiple chromosomes. Each D-genome chromosome was represented by two markers in the 14-member SSR panel. SSR loss was measured for each line of the 15-Gy plant panel and the 15- and 20-Gy endosperm panels on a per chromosome basis. The number of chromosomes with detected deletions was calculated for each line.

**Figure 3:**
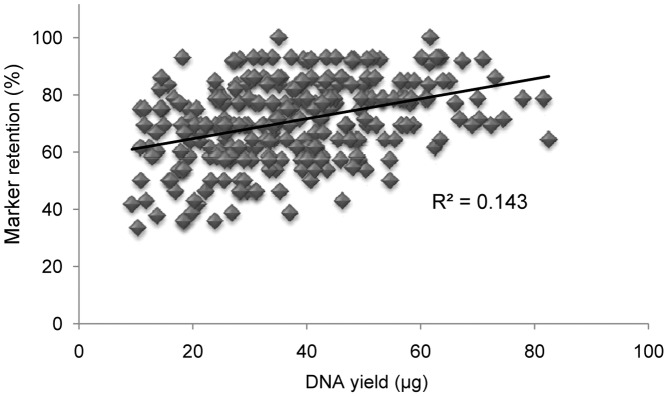
Correlation between marker retention and DNA yield. The total yield of DNA for 282 endopserm samples derived from 20-Gy treated pollen varied positively with the rate of marker retention (*p*<0.01). No lines were found with loss of all tested markers.

We compared the RH potential mapping of the 15-Gy plant, and 10-, 15- and 20-Gy endosperm DGRH_1_s based on the percentage of samples displaying marker retentions between 40 and 70%. Because of the relatively high rates of marker retention in the 10-Gy endosperm DGRH_1_s, that treatment was not characterized further. The percentage of lines retaining 40–70% of tested markers was significantly higher (*p*<0.01) in material derived from 15-Gy derived endosperm (23%) than that of 15-Gy derived plants (3%) (Figure 1). The 20-Gy endosperm DGRH_1_s had the highest percentage (∼50%) of lines with marker retention rates between 40 and 70%. In the 15-Gy plant DGRH_1_s, more than 45% of lines retained all tested markers whereas in the 15-Gy endosperm DGRH_1_s only 30% of lines retained all tested markers (Figure 1). However, only 11.4% lines retained all the tested markers in the 20-Gy endosperm DGRH_1_s. To create a genome-wide map in any species, it is resource-effective to develop germplasm harboring deletions in multiple chromosomes. Based on retention of the 14 SSR markers, we calculated the percentage of lines carrying breaks for multiple chromosomes **(** Figure 2 **)** in all three panels. In 15-Gy plant DGRH_1_s ∼19% of lines were estimated to carry no breaks for any D-genome chromosome whereas this measure was reduced to 5.8 and 1.0% in the 15- and 20-Gy endosperm panels, respectively. In the 15-Gy plant panel, a small percentage (∼4%) of lines were found to carry breaks in at least four chromosomes whereas 15% of lines in the 15-Gy endosperm DGRH_1_s lost markers in at least four chromosomes. The 20-Gy endosperm DGRH_1_s yielded the highest percentage of lines with breaks in four or more chromosomes (∼44%) and ∼19% of lines from that treatment carried deletions in five or more chromosomes. A correlation (*p<*0.01) between retention rates of the 14 SSR markers and DNA yield per line was observed based on 282, 20-Gy endosperm lines (Figure 3). The lowest marker retention measured of all lines tested was 33%.

### Mapping Panel Selection and Marker Retention

Our survey of SSR markers demonstrated the highest levels of chromosome deletions and multi-chromosome breaks in the 20-Gy endosperm DGRH_1_s. Because endosperm yields a finite quantity of DNA and we anticipated the need to utilize multiple marker platforms, total DNA yield became a factor for mapping panel selection. Endosperm tissues in seeds produced by pollinations with 15-Gy irradiated pollen yielded an average of 65.8 µg of DNA, whereas endosperm from 20-Gy treated pollen yielded an average of 49.6 µg of DNA. Not surprisingly, DNA yield was found correlated to endosperm size (data not shown). We chose lines that yielded more than 20.0 µg of DNA and exhibited marker retention ranges of 40–70%. From 282 20-Gy endosperm lines screened with 14 SSR markers, we selected 81 lines for high-throughput genotyping with the DArT marker platform. In addition to SSR and DArT markers, we assayed 13 D-genome specific ESTs, 5 insertion site-based polymorphism (ISBP) markers [35], and 10 repeat element junction (REJ) markers [36] on the selected 20-Gy endosperm panel by PCR to aid map anchoring.

Measured marker retentions for five different marker classes in the 81-member mapping panel are reported in Table 1. When the marker retention percentages of the different marker types were compared, we found the relative retention frequencies among these five groups to be heterogeneous (*p*<0.01). This may be due to higher-than-expected marker retention of EST markers. The average marker retention percentage across all marker systems for the 20-Gy endosperm mapping panel was 55.5%. When compared on a chromosome basis, marker retention rates were heterogeneous among D-genome chromosomes (*p*<0.01). This heterogeneity was likely due to higher than expected retention of chromosome 4D. We compared the marker retention rates of the 14 SSR markers to the retention rates for all marker systems for each line. A strong positive correlation (*p<*0.01) between the preliminary per line SSR marker retention rates and retention rates based on 965 markers (DArT, SSR, EST, REJ, and ISBP) suggested that a small number of markers in the initial screen was sufficient to detect lines ultimately useful for mapping.

**Table 1:** Retention frequencies (%) of five marker classes measured in 81 lines of 20-Gy derived endosperm.

Marker class	Number of markers	Retention (%)
DArT	867	53.8
SSR	70	68.0
EST*	13	79.2^a^
REJ	10	61.4
ISBP	5	63.9
Total	965	55.5^b^

a heterogeneous *(p = *0.002*)*,^ b^average across all markers.

### D-genome RH Mapping

DArT analysis yielded 867 markers present in Chinese Spring and absent in Altar 84. A total of 948 markers (867 DArT, 70 SSR, and 11 EST markers) were grouped using Carthagene “group” command at a LOD of 8 and a threshold distance of 50 cR. Initial grouping provided 41 linkage groups. Chromosomal locations of linkage groups were identified based on previously bin-mapped markers (http://www.cerealsdb.uk.net/dart/index.htm) and comparison with marker patterns in one of seven D–genome nullisomic-tetrasomic lines included in the assays. Precise loaction of four D-genome groups (based on nullisomic-tetrasomic data) was unavailable as none of the four groups included bin-mapped markers. Of the remaining 37 linkage groups, 12 were from A-genome chromosomes, 11 from B-genome chromosomes, and 18 belonged to D-genome chromosomes. Of 948 markers, 737 were assigned to D-genome chromosomes. Average marker retention percentages per line calculated for A-, B- and D-genome derived markers were 58.8, 58.9, and 54.7% respectively. On a per marker basis, we calculated relative retention frequencies of 54.4, 57.3 and 56.5% for A, B and D genomes respectively. These marker retention rates were homogeneous among the three genomes (*p* = 0.88). Markers from individual chromosomes were used to create a marker scaffold. The fewest markers (18) were found on 4D which has the lowest RH map distance. A total of 205 markers on chromosome 3D and 300 markers on chromosome 7D were grouped at LOD scores of 8 and 10, respectively. The lower marker density on chromosomes 1D, 2D, 5D, and 6D required relaxation of mapping parameters to calculate linkage: 1D (LOD 3), 2D (LOD 5), 4D (LOD 5), 5D (LOD 3) and 6D (LOD 5).

The D-genome RH map based on the 81-member endosperm panel defined 18 linkage groups with a total length of 5054.2 cR_2000._ Total map distance for individual chromosomes ranged from 181.1 (4D) to 1200.2 cR_2000_ (7D) (Table 2). Single linkage groups for entire chromosomes were not formed likely due to unequal distribution of DArT markers. A total of 354 unique loci were mapped using 737 markers. Numbers of markers, mapped loci, obligate breaks, unlinked markers (singletons), map lengths, and the two point LOD scores for each D-genome chromosome are reported in Table 2. Although locus orders in RH linkage groups were in general agreement with published maps, some differences were observed. For example, small inversions of marker order were observed on chromosomes 1DS, 3DL and 6DS relative to consensus genetic maps (Figures S1, S3, and S6). Chromosomes 3D (205 markers) and 7D (300 markers) contained the largest number of mapped markers.

**Table 2:** Characteristics of RH maps developed from 20-Gy derived endosperm.

Chromosome	Number ofmapped markers	Number ofmappedloci	Number oflinkagegroups	Number ofobligatebreaks	Singletons	Two Point LOD score Average (Range)	Map length (cR_2000_)
1D	62	49	2	225	5	14.9 (6.5–19.1)	918.5
2D	37	27	2	124	2	13.8 (8.9–18.9)	419.3
3D	205	87	2	331	2	16.7 (8.1–21.2)	1041.5
4D	18	10	4	36	2	16.2 (9.6–20.0)	181.1
5D	44	34	2	177	2	14.2 (3.8–21.7)	639.8
6D	60	42	2	190	3	14.9 (6.2–19.6)	653.8
7D	300	105	4	366	17	16.8 (9.7–22.3)	1200.2
total	737	354	18	1431	33		5054.2

### Mapping of Pericentromeric Region

Marker distribution along all seven D-genome chromosomes was uneven and the majority of markers mapped to distal chromosome bins (Figure S1, S2, S3, S4, S5, S6, and S7). Marker density in the pericentromeric bins was low relative to the terminal bins. To test the ability of the RH endosperm panel to map pericentromeric regions, we assayed 12 markers (EST and SSRs) previously mapped to 7D pericentromeric bins. Because of the paucity of available markers, we relaxed the grouping criterion to 100 cR_2000_ and a LOD score of 3.0 to create a lower confidence map of the bins flanking the 7D centromere. This map reproduced the order of the SSR markers in the consensus genetic map [37] (Figure S8). Bin locations of the EST markers also concurred with the calculated RH map. In regions of low recombination, it has been shown that tightly linked markers on a genetic map may be separated by large physical distances. For example, the distance between loci *Xwmc653* and *Xwmc221*on the consensus genetic map is 2.0 cM, however on the RH map they are separated by 212.2 cR_2000._ Similarly, SSR loci *Xgwm437* and *Xwmc221* are completely linked on the consensus genetic map but are 130.1 cR_2000_ distant on the endosperm RH map (Figure S8). Using SSR and EST markers we also created a RH map of the pericentromeric regions of chromosome 1D.

**Figure 4:**
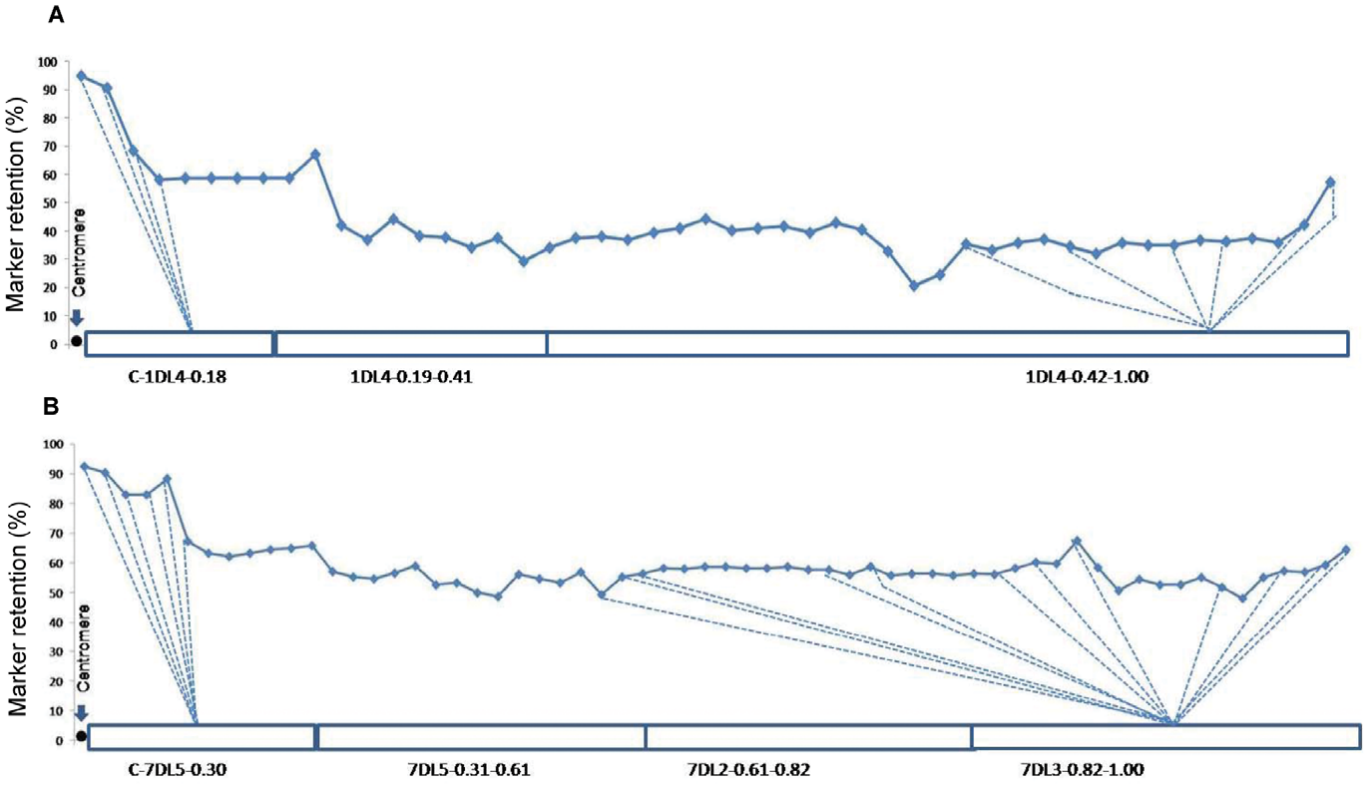
Marker retention along chromosomes 1DL and 7DL. Markers arranged in map order from the centromere (•) on the left to the telomere on the right; location of centromere estimated from other maps. Retention rates based on 81-member endosperm mapping panel. Equal spacing of markers in this representation does not represent physical location. Markers mapped to bins indicated by dotted lines.

When arranged in map order, markers flanking the centromere of chromosomes 1D and 7D showed higher retention than markers on distal regions (Figure 4). Marker retention across chromosome 7D was studied in detail and varied significantly (*p* = 0.01) between the distal bins (54.4%) and the pericentromeric bins (82.0%). Slightly higher marker retention was also observed towards the telomeres of chromosomes 1D and 7D (Figure 4). No heterogeneity of marker retention frequencies between distal bins of chromosomes 2D, 3D, 5D, and 7D was found (*p = *0.67).

### Calculated Map Distance Resolution Similar to Physical Distance Resolution Measurements

RH map resolution is a function of the number of lines and the number of chromosome breaks induced [8], [38]. In the present study we estimated resolution using markers mapped to 13 cytogenetic bins on six chromosomes with significant marker coverage totaling 1517.7 Mb (Table 3). The total RH map length of these bins was 4081.5 cR_2000._ One cR_2000,_ defined as the distance at which there is a 1% probability of a break between two markers, corresponded to ∼372.0 kb. Calculation of a ray (equivalent to 100 centirays) predicts an average fragment size of ∼37.2 Mb. The mapping potential (average frequency of chromosome breaks) of an RH panel can be estimated by dividing average fragment size by the number of genomes represented in the panel (81 lines×0.55 retention frequency = 44.6 X coverage) [8], [39]. Thus the average potential resolution (37.2 Mb/44.6 genomes) was calculated as ∼830 kb for the 81-member endosperm panel using information from six of the D-genome chromosomes. Map resolution was heterogeneous among the chromosomes, however this heterogeneity may be due to lower than expected resolution of 1DL and 2DS. Low marker density in some regions may have affected this calculation. Since resolution is a function of the number of lines, it can easily be increased by adding more panel members.

**Table 3:** Resolutions of D-genome RH maps for selected bins with high marker representation.

Chromosome	Chromosome bin	Bin size (Mb)	Bin map length (cR_2000_)	Mb/cR_2000_	Marker retention	Map resolution (Mb)
1DL	C-1DL-2-0.18-0.41 to 1DL-2-0.41-1.00	312.4	490.6	0.64	48.0	1.65
2DS	2DS-5-0.47-1.00	167.5	242.3	0.69	58.0	1.47
3DS	3DS-3-0.24-0.55 to 3DS-6-0.55-1.00	244.0	591.5	0.41	51.0	0.99
3DL	3DL-3-0.81-1.00	85.3	251.0	0.34	54.0	0.78
5DL	5DL-1-0.60-0.74 to 5DL-5-0.76-1.00	196.0	357.7	0.55	62.0	1.10
6DS	6DS-0.79 to 6DS-1.00	68.0	338.0	0.20	59.0	0.42
7DS	7DS-4-0.61-1.0	134.9	815.4	0.17	45.0	0.47
C-7DS - C-7DL	C-7DS-4-0.36 to C-7DL-5-0.30	241.0	813.60	0.30	56.0	0.66
7DL	7DL-3-0.82-1.00	68.6	181.4	0.38	58.0	0.81
means (SD)				0.41 (0.17)	54.6 (5.25)	0.93 (0.40)
95% confidence interval				0.28–0.54	50.5–58.6	0.62–1.23
totals		1517.7	4081.5	0.37^a^	55.0^b^	0.83^a^

a calculated from totals, ^b^calculated across all bins.

The majority of 7D markers mapped to deletion bins which account for ∼39% of the short arm of chromosome 7D and are estimated to include ∼135 Mb. Calculating as above, the mapping resolution potential was 470 kb for bin 7DS-4 (Table 3) similar to the overall average. The average map resolution (∼660 kb) of the pericentromeric region (bins C-7DS-4 to C-7DL-5) was similar to the calculated resolution of the terminal bins. We calculated cR_2000_/cM ratio of 18.1 for 7DS using markers *wmc506* and *wmc698* which are common to the consensus genetic maps and the RH maps for 7DS (Figure S7). The cR_2000_/cM ratio of the pericentromeric region of 7D calculated using markers *wmc653* and *wmc488* (Figure S8) was 84.1.

The estimates of mapping resolution presented above rest on positions of markers based on calculated maps. To directly test our predictions of resolution, we determined the number of breaks occurring between three 3B markers, *cfp5018*, *cfp5034* and *cfp5036* [35], separated by 685 kb and 56 kb, respectively. From 81 lines tested, we found two breaks between *cfp5018* and *cfp5034*. We detected no breaks between closely spaced markers *cfp5034* and *cfp5036*. This suggested that markers as close as ∼685 kb could be resolved using this panel in agreement with the map-based calculations.

### Size and Position of Deletions

Two terminal bins on chromosomes 3DS and 7DS had high numbers of well-spaced markers and well-populated maps were calculated (Figure S3 and S7). Using the calculated Mb/cR_2000_ ratio for each bin (Table 3), we estimated the physical distance between each marker. With marker data arranged in map order for each line, deletions were counted and physical size of each deletion estimated. Using 157 markers, 143 deletions were defined on 3DS. Similarly 237 markers defined 161 deletions on 7DS. Thirty eight percent (54/143) of all deletions were terminal on 3DS. Similarly, 32% (51/161) of the 7DS deletions were terminal. The remainders, approximately two thirds in each case, were interstitial deletions. The average number of interstitial breaks per bin per line was 1.23 and 1.38 for terminal bins of 3DS and 7DS respectively. The number of interstitial deletions per line ranged from 0 to 5 for 3DS and 0 to 7 for 7DS.

**Figure 5:**
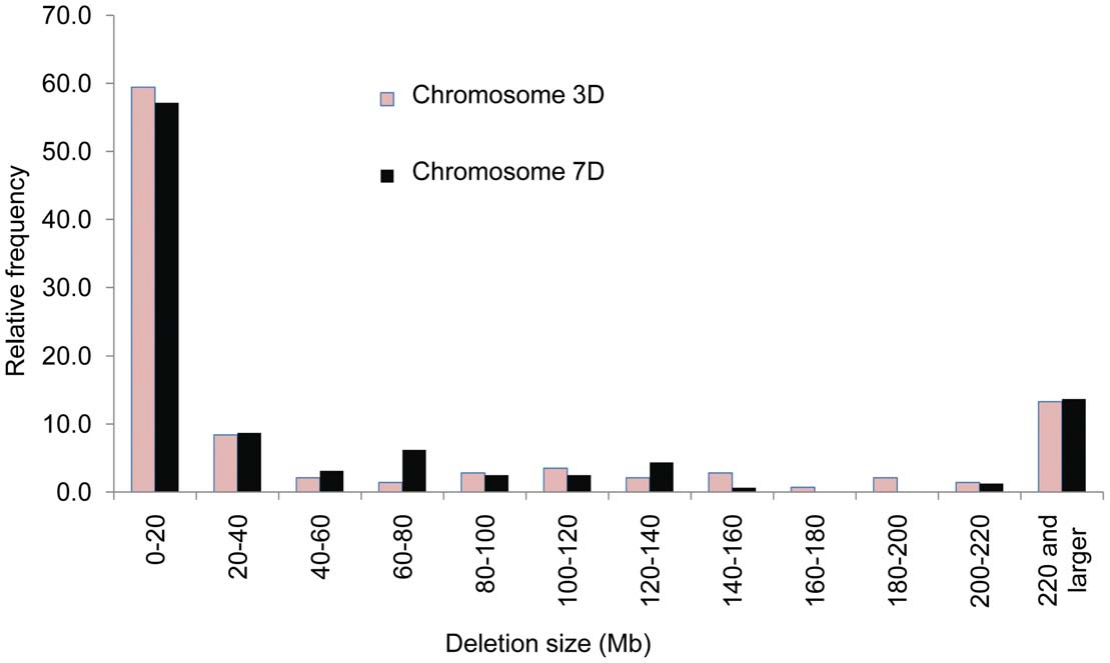
Distribution of sizes of the radiation-induced deletions. Deletion sizes were calculated from four distal bins of chromosomes 3D and 7D. Average distance between markers was calculated by dividing physical size of bins (Mb) by length of RH map. Size of deletions was calculated manually from the number of markers lost in each deletion.

Figure 5 displays a histogram of the relative frequencies of the 3DS and 7DS deletions. The highest proportion of deletions (59.4% for 3DS and 57.1% for 7DS) in both chromosomes were <20 Mb. However a number of larger deletions were also observed; in both chromosomes approximately 13% of the deletions were 220 Mb or larger. Distribution of deletion sizes were similar (*p = *0.11) between chromosome 3D and 7D. Further analysis of deletions <20 Mb revealed that 44.2% of 3DS deletions and 46.3% of 7DS deletions were 5 Mb or less. Combining the data from both chromosomes, ∼20% of the smallest deletions were 5–10 Mb in size with the remainder of that class in the 10–20 Mb intervals.

**Figure 6:**
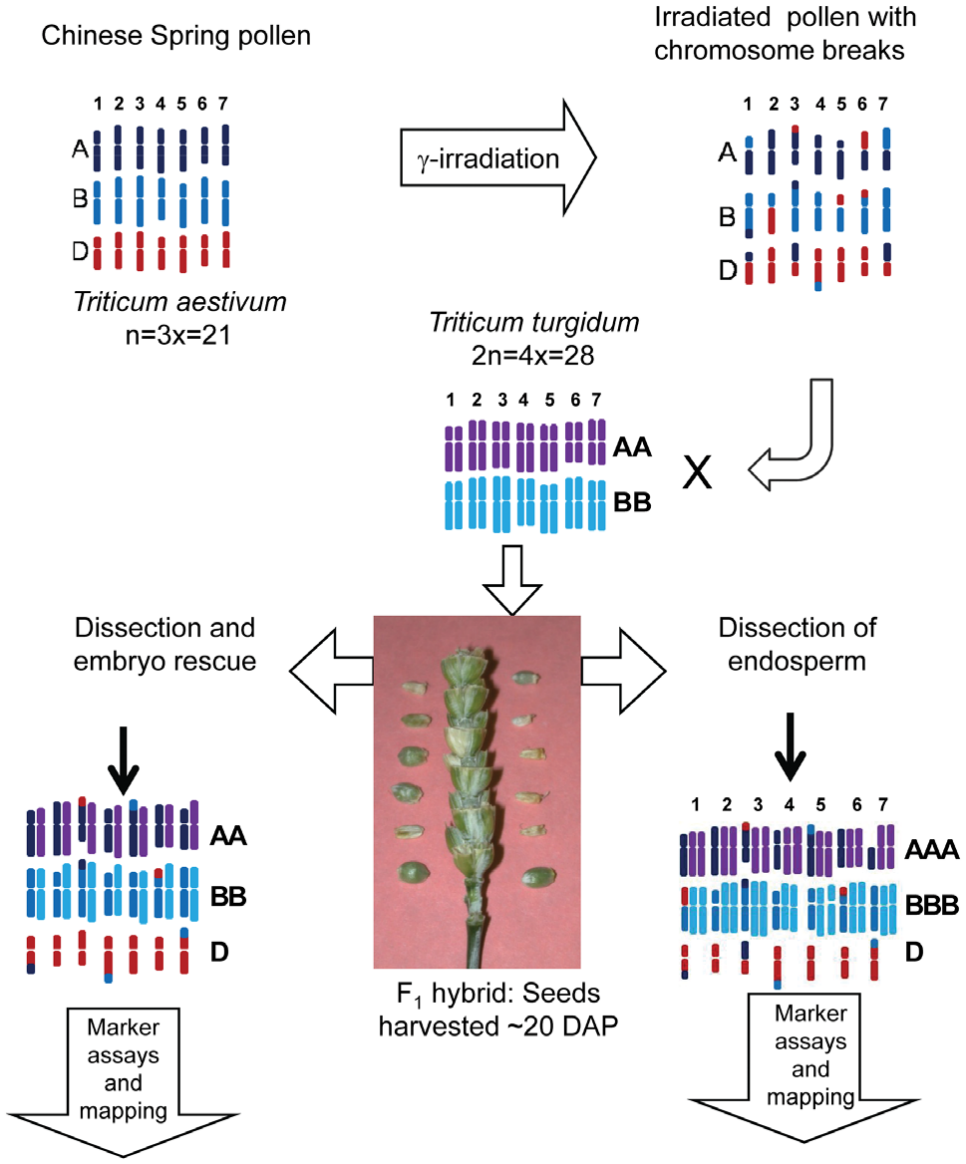
Schematic for making D-genome radiation hybrids (DGRH_1_s). Dehiscent spikes of hexaploid wheat variety Chinese Spring (*Triticum aestivum* 2n = 6× = 42, AABBDD) were cut from flowering plants and γ-irradiated. Pollen from irradiated spikes was immediately used to pollinate emasculated tetraploid wheat variety Altar 84 (*T. turgidum,* 2n = 4× = 28, AABB*)*. Seeds of F_1_ hybrids were harvested ∼20 days after pollination (DAP), and from each seed embryo and endosperm were dissected. Embryos were transferred to rescue media to recover plants whereas DNA was directly extracted from endosperms. In this system DNA from each embryo-derived plant and endosperm contain independent mutation events.

Across seven chromosomes, we detected 1431 obligate breaks (Table 2). The number of obligate breaks increased with the number of markers applied in a ratio that ranged from 3.5 breaks/marker for 7D to 4.7 breaks/marker for 1D. Chromosomes 3D and 7D, with the highest number of markers, contained the highest number of obligate breaks.

### Marker Estimate to Saturate the Maps

RH map resolution is a function of average frequency of chromosome breaks and number of lines, but can only be realized if sufficient markers are available. Resolution of other RH maps is estimated to range from 0.45–1.32 Mb [8], [38]. The average resolution we calculated for nine D-genome regions (Table 3) was ∼830 kb, well within the range of the studies cited. Extrapolating that estimate to the ∼5 Gb D-genome, we estimate there are approximately 6,025 break-point-defined bins present in the 20-Gy endosperm panel. Assuming that radiation-induced chromosome breaks are induced randomly, the number of randomly distributed markers necessary to label each bin (*p* = 0.95), can be modeled with a Poisson distribution. We estimate that ∼18,000 markers would be required to fully utilize the potential of the RH endosperm mapping panel.

## Discussion

Pollen irradiation provided an efficient means of inducing chromosome breaks in plants. D-genome radiation hybrids produced by γ-irradiation of pollen avoid complications associated with seed irradiation or *in vitro* cultures. Seed irradiation creates a genetically heterogeneous population of cells yielding chimeric plants [40]. In wheat, like other angiosperms, tolerance of hypoaneuploidy is much higher in diploid sporophytes than haploid gametophytes and wheat sporophytes are shielded by genome buffering. Although tolerated in irradiated-seed derived plants, aberrant chromosomes may be lethal in megagametophytes or microgametophytes and transmission highly reduced [41], [42], [43]. Use of pollen irradiation at anthesis minimizes selection pressure against hypoaneuploidy. Xu et al. [44] reported that pollen development is exclusively determined by the vegetative nucleus and that aneuploidy or hypoaneuploidy of the sperm nuclei has little effect on pollen phenotype. Therefore gametophytic aneuploidy is more likely to be transmitted if induced *de novo* following gametophyte development from a normal sporophyte rather than from uniformly aneuploid sporophytes [20], [44]. The ability to induce chromosome breakage after the final microgametophyte mitotic divisions, and thereby avoid meiotic sieving, likely explains our ability to generate tissue harboring significantly more deletions than seed irradiation methods [22].

### Endosperm Development is Less Affected by Radiation-induced Aneuploidy

The lower survival rate of embryos relative to endosperm for each treatment suggests that paternally derived genetic deficiencies tolerated by developing endosperm may be lethal to developing embryos. One likely explanation is that genetic buffering provided by triploid endosperm compensates for unbalanced gene dosage when a copy of an essential gene was deleted. Similarly, Vizir and Mulligan [45] reported that deletions which produced lethal gene-dosage imbalances in heterozygous diploid progeny could be overcome by using diploid eggs. Alternatively, the less complex endosperm may require fewer essential genes for development than embryos. Similar to other reports [27], we found decreased size and endosperm weight correlated with increased radiation dosage and no endosperm development with pollen treated with more than 25 Gy. Although development of autonomous endosperms (development of endosperms without fusion of male gametophyte) was reported by others as fertilization independent seeds (*fis*) [46], [47], we did not find any autonomous endosperm development as a consequence of sub-lethal irradiation of the male gametophyte. This agrees with the report of Aw et al. [48] wherein fusion of a male gametophyte is absolutely required for endosperm development in *Arabidopsis*.

### Advantages of Pollen-irradiation Endosperm Mapping Panels

Radiation hybrid mapping panels generated by cell-fusion protocols used in animal systems struggle against low marker retention rates [49] whereas the protocols used in RH mapping projects of plants produce lines with significantly higher average marker retention rates than reported here. For example average marker retention rates of 75–85%, 87–94%, and 97% have been reported in RH panels of maize, cotton and wheat, respectively [17], [18], [19], [22]. The technique of pollen irradiation was utilized for RH mapping in plants by Gao et al. [19]. They reported a wide-cross whole-genome radiation hybrid (WWRH) system for cotton, based on γ-irradiation of *Gossypium hirsutum* L. pollen crossed with a double haploid line of *Gossypium barbadense* L. However, the efficiency of this method was limited by the requirement for polymorphic markers and high marker retention frequencies. In wheat, the problem of marker polymorphism can be overcome by judicious use of parents to place the target genome in monosomic condition in the hybrid generation [22]. We overcame the high marker retention rates reported in that work by coupling the pollen irradiation method and utilization of the radiation-tolerant endosperm.

An advantage of the pollen irradiation system is that each irradiated hexaploid spike can fertilize multiple emasculated tetraploid ovules, and each tetraploid spike can yield 10 to 20 quasi-heptaploid endosperm tissues. Because endosperm was harvested ∼20 DAP, the time needed to optimize radiation treatments and develop a panel was shortened significantly. Screening with a small number of SSRs provided an accurate and quick measure of the mapping utility of each treatment and simultaneously identified the most informative lines thereby minimizing the cost of final genotyping. From about 4 to 20 DAP, endosperm undergo a mitotic cell proliferation phase followed by an endoreduplication phase [50]. Endoreduplication during endosperm development seems to be a universal mechanism in cereal crops, and is strongly correlated with high DNA content and increased cell size [51], [52], [53]. The programmed amplification of DNA in endosperm greatly enhanced its utility for RH mapping. The development of high throughput marker genotyping systems [32], [54], [55], [56] will increase our ability to type thousands of markers on quickly produced endosperm RH panels. The total DNA recovered from endosperm samples in this study ranged from 9.3 to 82.5 µg. Most existing high throughput genotyping platforms require small amounts of input sample DNA. For example the DArT analysis performed for this study required only 0.5–1.0 µg DNA (http://www.triticarte.com.au/content/FAQ.html). Other high throughput platforms such as NimbleGen (Roche NimbleGen, Madison, WI) or Infinium (Illumina, San Diego, CA) require only 1 ug or less DNA to assay tens of thousands of markers. (http://www.nimblegen.com/products/lit/05434483001_NG_CGHLOH_UGuide_v9p1.pdf http://www.illumina.com/technology/infinium_hd_assay.ilmn). Therefore, this system provides ample resources for genotyping.

A common problem in meiotic mapping is the inability to map the pericentromeric regions of chromosomes, which accounts for 40 to 50% of their physical size, due to lack of recombination [57], [58]. We imagined that most paternal DNA is carried, both in embryos and endosperm, as centromere-dependent chromosomes although translocations into maternal chromosomes may occur as well. Therefore higher retention of markers near functional centromeres was expected. Nonetheless, we were able to map the pericentromeric region of chromosome 7D demonstrating that sufficient breaks occur in this region to make them amenable to RH mapping. Radiation-induced chromosome breaks were expected to be randomly induced, and therefore the physical size represented by a cR_2000_ would remain relatively constant. As expected, the Mb/cR_2000_ ratio was relatively consistent among all six D-genome chromosomes analyzed including both distal and proximal regions of the chromosome. This is the expected advantage of RH maps over genetic maps for the anchoring of contigs in regions of low recombination. Proof of this will require further genotyping of this panel with greater numbers of evenly distributed markers. The fact that no endosperm samples were recovered with less than 33% marker retention suggests the possibility that regions of the paternal genome required for endosperm development might be difficult to map in this manner. Conversely, one might be able to identify regions of the genome important for endosperm development in exactly this manner.

Approximately 94% of DArT markers were restricted to distal bins of the chromosome arms and representation in pericentromeric bins was negligible. EST-based high-density physical maps of wheat chromosomes reveal ∼85% of wheat genes are present in small regions spanning 5%–10% of the chromosome at the distal ends [59], [60], [61], [62]. DArT markers were developed to target these hypomethylated gene-rich regions of chromosomes and their distribution on our maps was predictable. The distribution of markers across linkage groups in our study likely reflects the distribution of available markers not the mapping potential of the RH endosperm panel. Clustering of DArT markers in our study, similar to reports in DArT genetic maps [31], [33], is likely due to the presence of redundant clones in the genomic representation.

To make resolution calculations we assumed markers were distributed over the entire length of bins as exact positions are unknown. This is likely an overestimate of the physical size of the region defined by the markers. Therefore our estimates of resolution are conservative and may underestimate the mapping potential of the panel

### RH Mapping Reveals Patterns of Radiation-induced Deletions in Pollen

RH mapping of endosperm-carried deletions gave a unique opportunity to estimate the frequency and size of post-meiotic radiation-induced deletions in wheat. Double strand break (DSB) repair by non-homologous end joining (NHEJ) is inherently mutagenic and would therefore appear under selection pressure in pollen. However, once the final division forming the two sperm cells has occurred, no opportunity for homologous repair remains leaving only the NHEJ pathway. We measured retention rates of the 14-SSR panel in 94 samples of endosperm and embryos derived from 15 Gy-treated pollen paired by recovery from the same seed. We found similar rates of marker retention in the paired samples but independent deletion events (data not shown). This confirms that radiation-induced damage had occurred after the final division as expected but says little about where the repair occurs.

Deletions sufficient in size to encompass at least two markers are required for RH mapping programs in order to detect linkage. Seed-irradiation experiments have reported many deletions <100 kb [63]. However pollen irradiation experiments in *Arabidopsis* that measure mutations in the F_1_ generation [64] produce large deletions (>2.0 Mb); the majority of these are not transmitted and would be lost in subsequent generations. Because of the genomic buffering inherent in the wheat genome and the simplified function of the endosperm, we likely recovered more deletions than would be viable in a diploid plant system like *Arabidopsis*. While radiation-induced deletions in plants are influenced by tissue and method of production, their characterization is influenced by the detection methods employed. Very small deletions were not detected in our system due to the markers utilized and map calculation, but are expected to be abundant. A higher density marker system (under development) will provide much better resolution and reveal smaller deletions. Nonetheless, an interesting pattern, in which the majority of deletions are <20 Mb, suggests an attractive route to fine-scale gene mapping. This would be particularly useful in chromosomal areas of low recombination. Although terminal deletions might be expected to predominate, the average number of deletions per arm (2.4) makes this unlikely. This is in agreement with other pollen-irradiation experiments using *Arabidopsis* [45] that found interstitial breaks most common.

The abundance of deletions induced by DSB repair in pollen and the tolerance of endosperm for aberrant paternal DNA provided a very useful and quickly-developed source of physical mapping information in wheat. As new marker genotyping platforms become available, this material can be utilized for the typing of different classes of marker to increase map density. DNA samples produced in this study will be available to the scientific community for typing more markers using high-throughput genotyping platforms. By simultaneous screening of wheat BAC libraries with RH-mapped markers, BAC contigs can be aligned to chromosome scaffolds irrespective of presence or absence of recombination. With sufficient markers, we will be able to build a physical scaffold with sufficient resolution to order BAC contigs prior to sequencing.

## Materials and Methods

### Plant Material

Hexaploid wheat line Chinese Spring (*Triticum aestivum*; 2n = 6× = 42; AABBDD), a standard genotype for cytogenetics, genetics, and genomics research, was used as the male parent. The tetraploid cultivar Altar 84 (*Triticum turgidum*; 2n = 28; AABB) was used as the female parent in all crosses.

### Production of Radiation Hybrids

To generate irradiated pollen, Chinese Spring (CS) plants were grown to flowering, and dehiscent wheat spikes were excised from the plant with stems kept in water. Entire spikes were irradiated with γ-rays and pollen from the irradiated spikes was immediately used to pollinate previously emasculated spikes of Altar 84 (Figure 6). All irradiation was performed in a Gamma Cell 220 irradiator (Gamma Irradiation Facility, Radiation Center, Oregon State University).

F_1_ hybrid seeds (DGRH_1_s) were harvested ∼20 days after pollination (DAP) and embryo and endosperm were dissected from each seed. Embryos were transferred to rescue media to recover plants whereas endosperms were stored at −20°C until used for DNA extraction. F_1_ embryos from a cross between these genotypes were quasi-pentaploid (AABBD) while F_1_ endosperm were quasi-heptaploid (AAABBBD).

### Characterization of DGRH_1_s

DNA extraction from plant (15-Gy DGRH_1_s) and endosperm tissue (10-, 15-, and 20-Gy DGRH_1_s) was performed using a modified version of the method described by Riera-Lizarazu et al. [17]. Embryos were dissected away from endosperm and endosperm placed in individual microtubes (Qiagen, Valencia, CA). Approximately 50 mg of tissue harvested from rescued embryos was also placed in individual microtubes. A tungsten bead (Qiagen) and 450 µl of lysis buffer (10 mM Tris-HCl pH 9.5, 10 mM EDTA, 100 mM KCl, 500 mM sucrose, 4 mM spermidine, 1.0 mM spermine, 0.1% (v/v) 2-mercaptoethanol, and 2.0% (w/v) sarkosyl) was added to each tube prior to grinding by two 90-second-cycles at 30 hertz with a Retsch MM300 mixer mill (Newton, PA). Samples were extracted one time with 300 µl phenol/chloroform (1∶1), precipitated with two volumes of 100% ethanol, and washed one time with 70% ethanol. The DNA pellets were resuspended in 100 µl TE (10 mM Tris-HCl ph 8.0, 1.0 mM EDTA). DNA samples were quantified using a NanoDrop ND-1000 UV-Vis Spectrophotometer (Thermo Scientific, Waltham, MA) and the integrity of the DNA was verified by electrophoresis on a 0.8% agarose gel. DNA extracted from endosperm tissues showed single high-molecular-weight bands (Figure S9). Plant and endosperm DGRH_1_s were characterized by assaying for loss of individual members of a set of 14 D-genome specific SSR markers, one per chromosome arm: *Xbarc149, Xcfd282, Xgwm261, Xbarc228, Xcfd141, Xwmc552, Xwmc818, Xwmc622, Xcfd18, Xcfd86, Xcfd49, Xcfd37, Xwmc506,* and *Xgdm150*. D-genome specificity of the markers was confirmed by comparison of amplification products produced from the tetraploid Altar with products amplified from the hexaploid Chinese Spring. Polymerase chain reaction (PCR) assays were carried out in a volume of 10 µl as described by Riera-Lizarazu et al. [22]. Because D-genome chromosomes in DGRH_1_s are carried in a monosomic condition, the assay detects marker loss by presence or absence of a marker-specific amplification product. To control for failed PCR reactions, an additional primer pair, AWJL3 [65] was multiplexed in all PCR reactions. Marker retention was expressed as the proportion of instances where a given marker was retained relative to the number of lines tested in a panel of individuals [22]. A set of non-irradiated (0-Gy) control endosperm tissue was also generated and DNA was extracted. The 0-Gy endosperm samples (*n* = 30) together with CS and Altar 84 control endosperm DNAs were tested with same set of 14 SSR markers as controls.

### Genotyping

Of 282 endosperm samples screened with markers, an 81-member subset with retention rates of the 14 SSR markers between 40% and 70% per line was selected for analysis and mapping. By preliminary selection, average marker retention rate of the endosperm mapping panel was reduced from 68% in the initial lines to ∼56% in the final panel. To aid grouping of markers, DNA was prepared from leaf tissue of seven nullisomic-tetrasomic Chinese Spring lines, each deficient for one of the seven D-genome chromosomes: N1D-T1A, N2D-T2A, N3D-T3A, N4D-T4B, N5D-T5A, N6D-T6A, and N7D-T7A. DNA samples were assayed by Triticarte Pty. Ltd. (Canberra, Australia).

### Radiation Hybrid Mapping

Grouping and ordering of RH marker data were carried out using the software package Carthagene [66]. It utilizes an equal-retention model and computes all two-point distance scores. Linkage groups were determined using minimum two-point LOD scores of 3.0. Markers which localized to specific D-genome chromosomes based upon absence from individual nullisomic-tetrasomic lines but did not group at this LOD score were counted as singletons. The Carthagene command ‘mapocb’ was used to calculate the number of obligate breaks.

### Statistical Analysis

The χ^2^ contingency test was performed to discern differences between effects on seed set and survival rate of embryos and endosperms from different radiation treatments. To test for differences in radiation-induced marker loss between radiation hybrids from the 10-, 15- and 20-Gy treatments, we assayed the panels with 14 SSR markers and performed χ^2^ contingency tests. The χ^2^ test of homogeneity was performed to detect differences in marker retention between the five types of markers used in this study (Table 1). A χ^2^ test of homogeneity was used to assess radiation-induced chromosome breakage differences among A-, B-, and D-genomes and among D-genome chromosomes. The same test was also used to assess whether or not radiation-induced chromosome breakage was homogenous within a given D-genome chromosome and to compare distribution of marker retention frequencies in proximal, pericentromeric, and distal bins of a given D-genome chromosome. Homogeneity of the distribution of deletion sizes among the two bins of chromosome 3D and 7D analyzed was also analyzed with a χ^2^ test.

## Supporting Information

Figure S1 **Radiation hybrid map for chromosome 1D.** Comparison of deletion bin map (Bin), radiation hybrid map generated in this study (RH), and consensus genetic map (Genetic) of chromosome 1D. Distances on RH map are in centiRays and on genetic map are in centiMorgans. Markers mapped to multiple maps are connected by dashed lines. Due to lack of markers from the centromeric regions, the RH maps formed two linkage groups.
(TIF)

Figure S2 **Radiation hybrid map of chromosome 2D.** Map comparison of chromosome 2D as described in Figure S1.
(TIF)

Figure S3 **Radiation hybrid map of chromosome 3D.** Map comparison of chromosome 3D as described in Figure S1.
(TIF)

Figure S4 **Radiation hybrid map of chromosome 4D.** Map comparison of chromosome 4D as described in Figure S1.
(TIF)

Figure S5 **Radiation hybrid map of chromosome 5D.** Map comparison of chromosome 5D as described in Figure S1.
(TIF)

Figure S6 **Radiation hybrid map of chromosome 6D.** Map comparison of chromosome 6D as described in Figure S1.
(TIF)

Figure S7 **Radiation hybrid map of chromosome 7D.** Map comparison of chromosome 7D as described in Figure S1.
(TIF)

Figure S8 **Radiation hybrid map of 7D pericentomeric region.** Comparison of deletion bin map, RH map, and consensus genetic map of the pericentomeric region of wheat chromosome 7D. Eight ESTs and four SSRs were mapped to the low-recombination pericentromeric region of chromosome 7D. Dotted lines connect markers mapped to multiple maps.
(TIF)

Figure S9 **DNA recovered from endosperm is high molecular weight.** Aliquots of endosperm DNA derived from 20-Gy-irradiated pollen were assayed by electrophoresis on a 0.8% agarose gel and visualized by staining with ethidium bromide. Lane 1 is unirradiated CS endosperm, lane 2 is unirradiated Altar endosperm and lanes 3 through 8 are 6 independent CS/Altar F_1_ endosperms. Numbers on right represent base pairs.
(TIF)
