# Supplementary figures and images for: Endosperm Tolerance of Paternal Aneuploidy Allows Radiation Hybrid Mapping of the Wheat D-Genome and a Measure of γ Ray-Induced Chromosome Breaks
Source: PLoS One. 2012 Nov 7;7(11):e48815. doi: 10.1371/journal.pone.0048815 (PMC3492231; doi:10.1371/journal.pone.0048815)

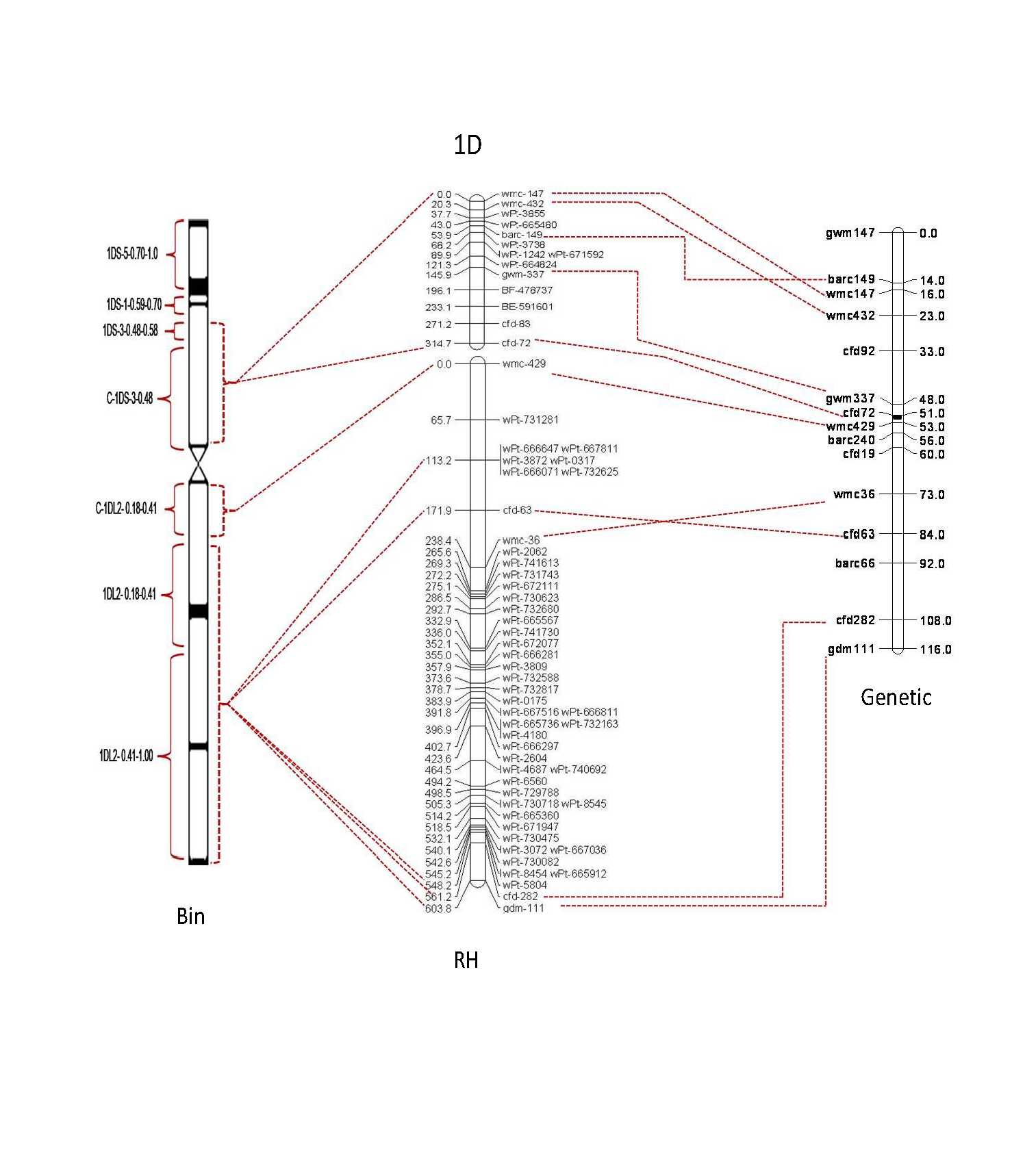

Supplement: Figure S1 — Radiation hybrid map for chromosome 1D. Comparison of deletion bin map (Bin), radiation hybrid map generated in this study (RH), and consensus genetic map (Genetic) of chromosome 1D. Distances on RH map are in centiRays and on genetic map are in centiMorgans. Markers mapped to multiple maps are connected by dashed lines. Due to lack of markers from the centromeric regions, the RH maps formed two linkage groups. (TIF) [file pone.0048815.s001.tif]

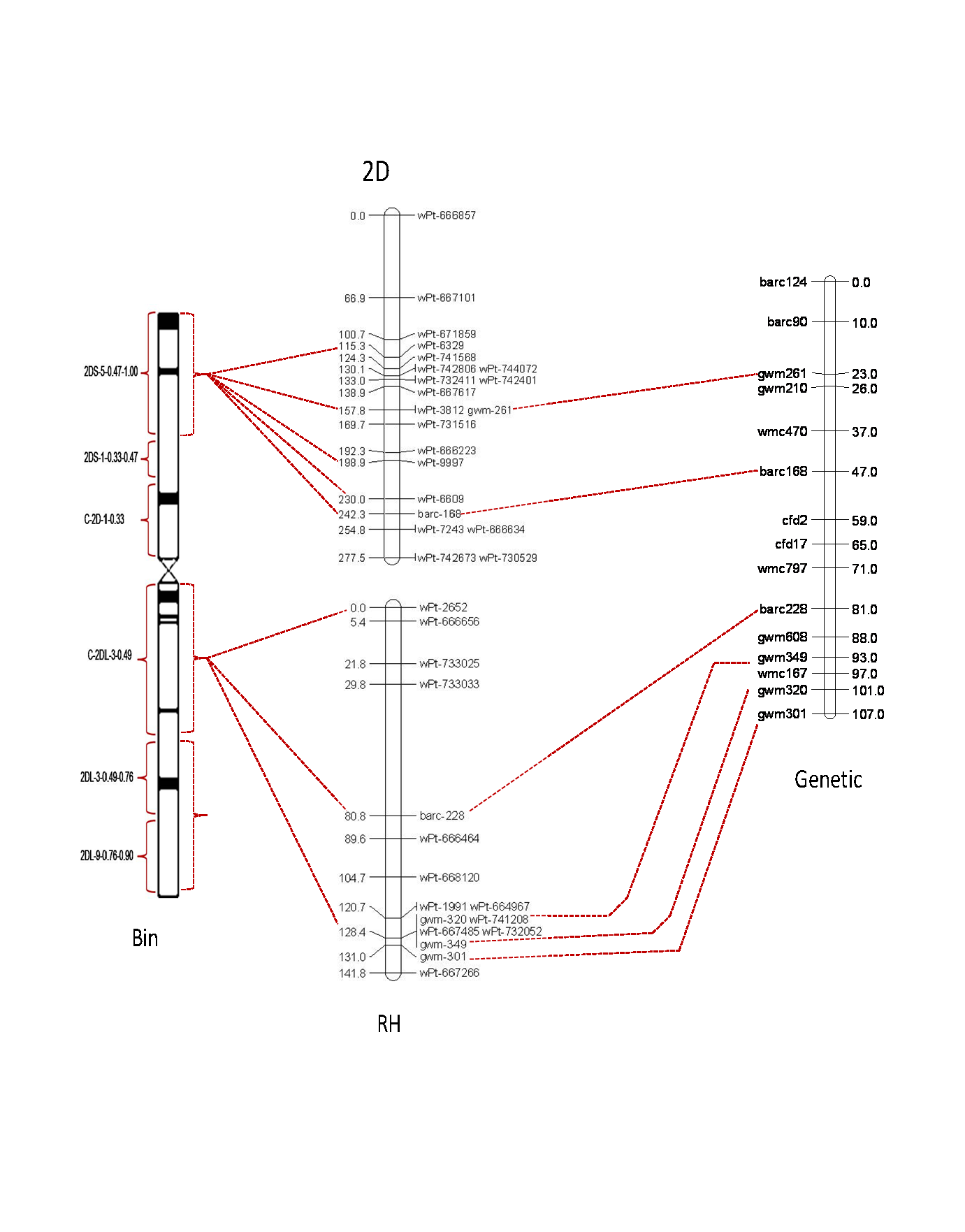

Supplement: Figure S2 — Radiation hybrid map of chromosome 2D. Map comparison of chromosome 2D as described in Figure S1. (TIF) [file pone.0048815.s002.tif]

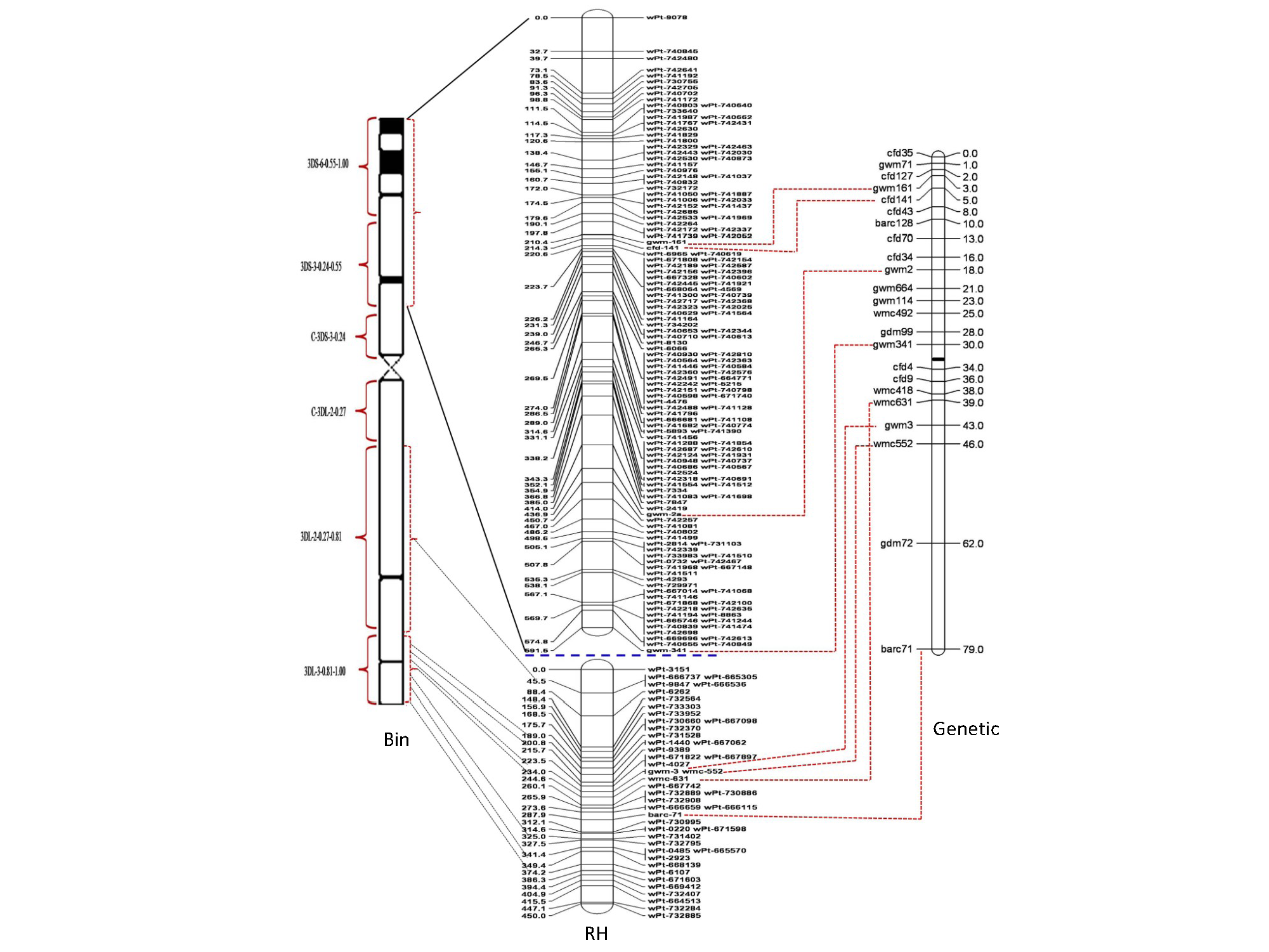

Supplement: Figure S3 — Radiation hybrid map of chromosome 3D. Map comparison of chromosome 3D as described in Figure S1. (TIF) [file pone.0048815.s003.tif]

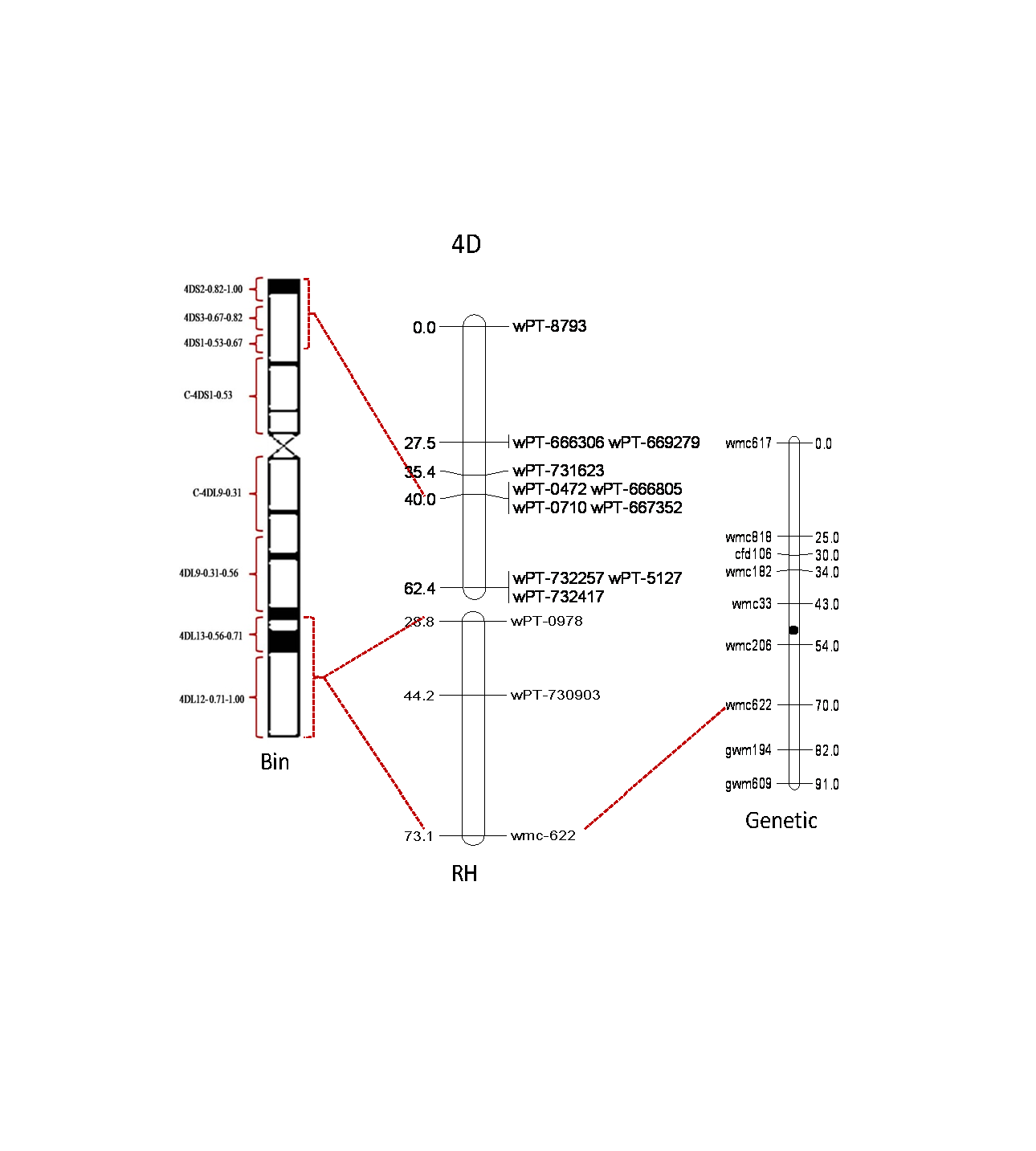

Supplement: Figure S4 — Radiation hybrid map of chromosome 4D. Map comparison of chromosome 4D as described in Figure S1. (TIF) [file pone.0048815.s004.tif]

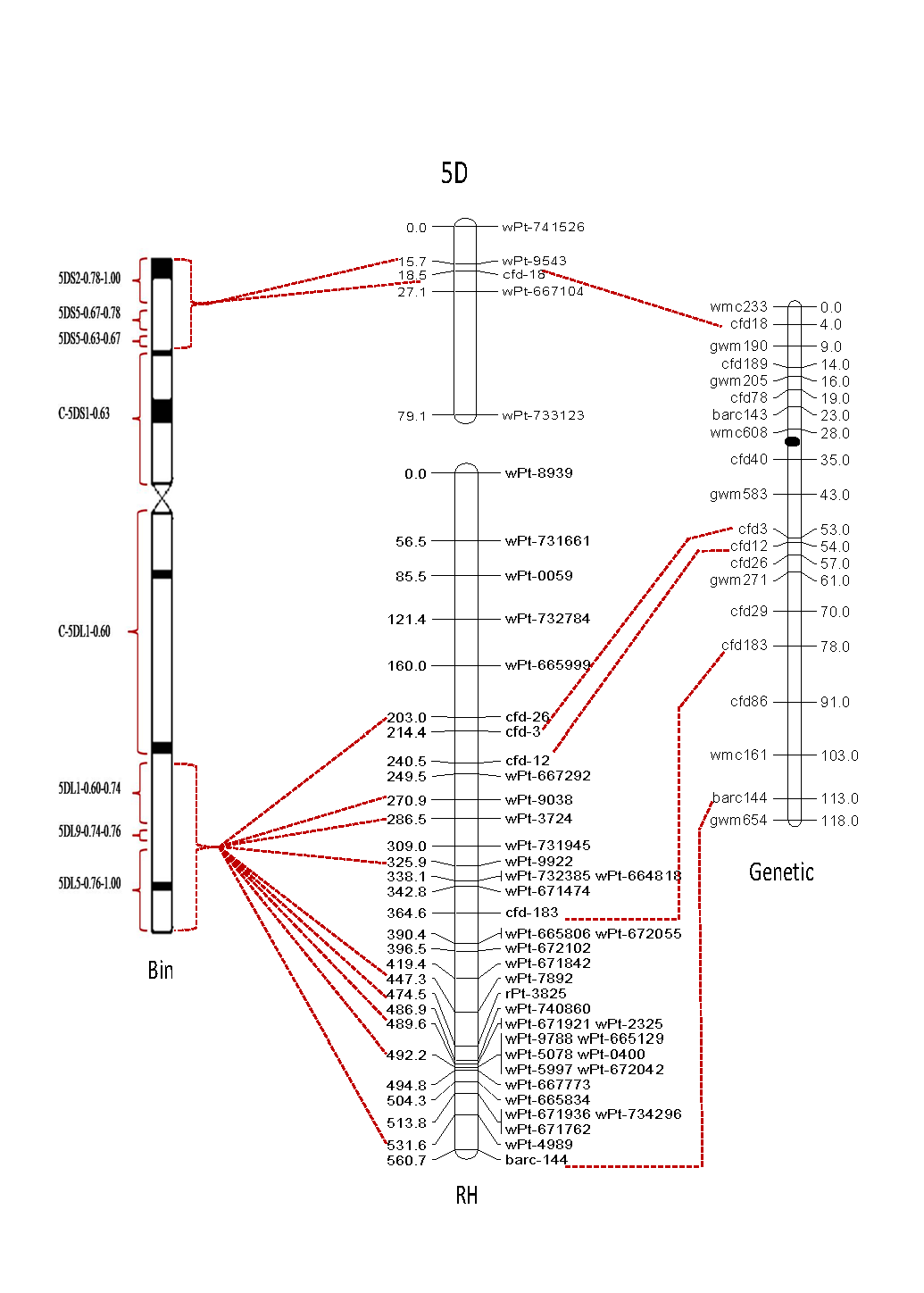

Supplement: Figure S5 — Radiation hybrid map of chromosome 5D. Map comparison of chromosome 5D as described in Figure S1. (TIF) [file pone.0048815.s005.tif]

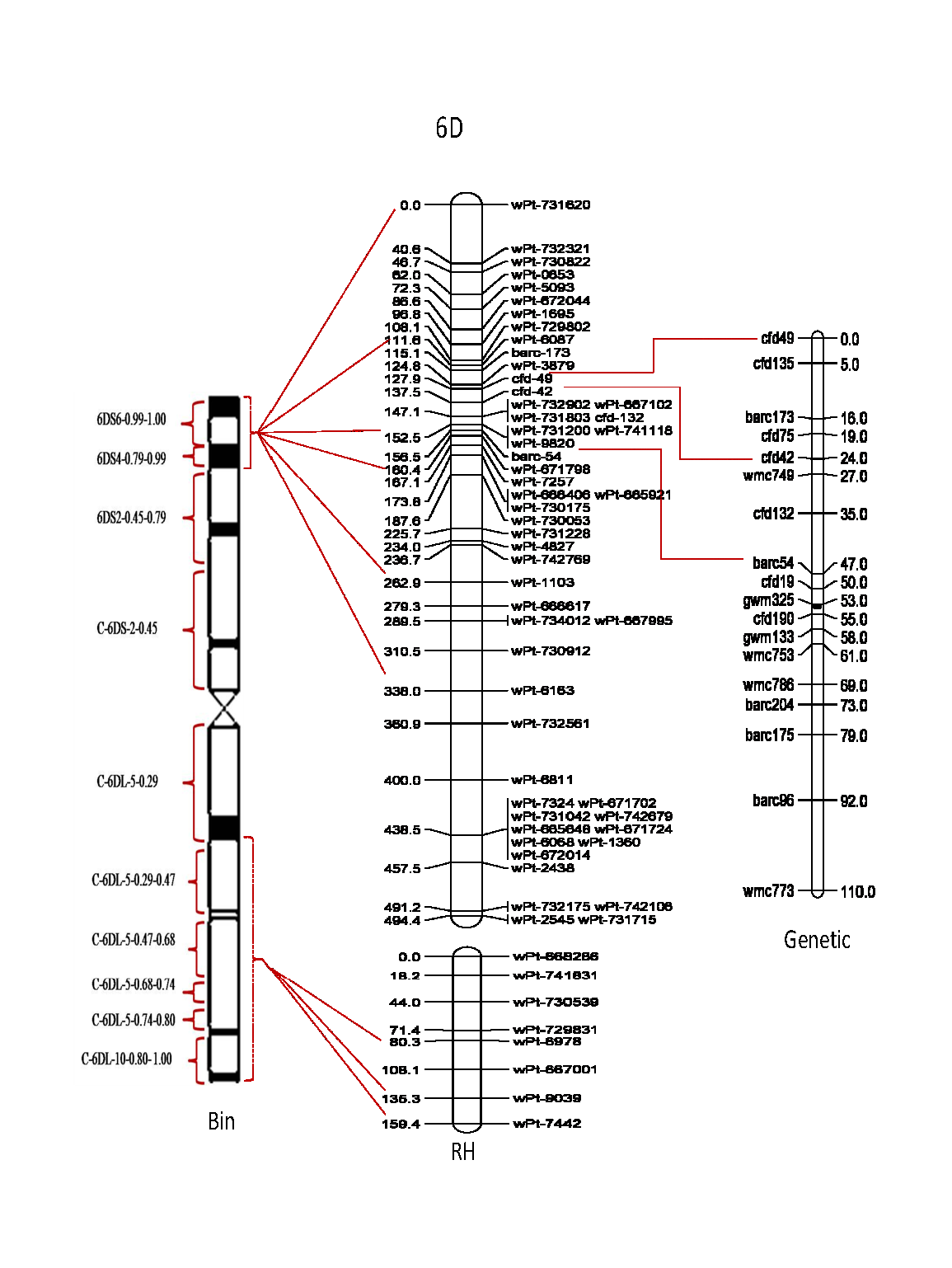

Supplement: Figure S6 — Radiation hybrid map of chromosome 6D. Map comparison of chromosome 6D as described in Figure S1. (TIF) [file pone.0048815.s006.tif]

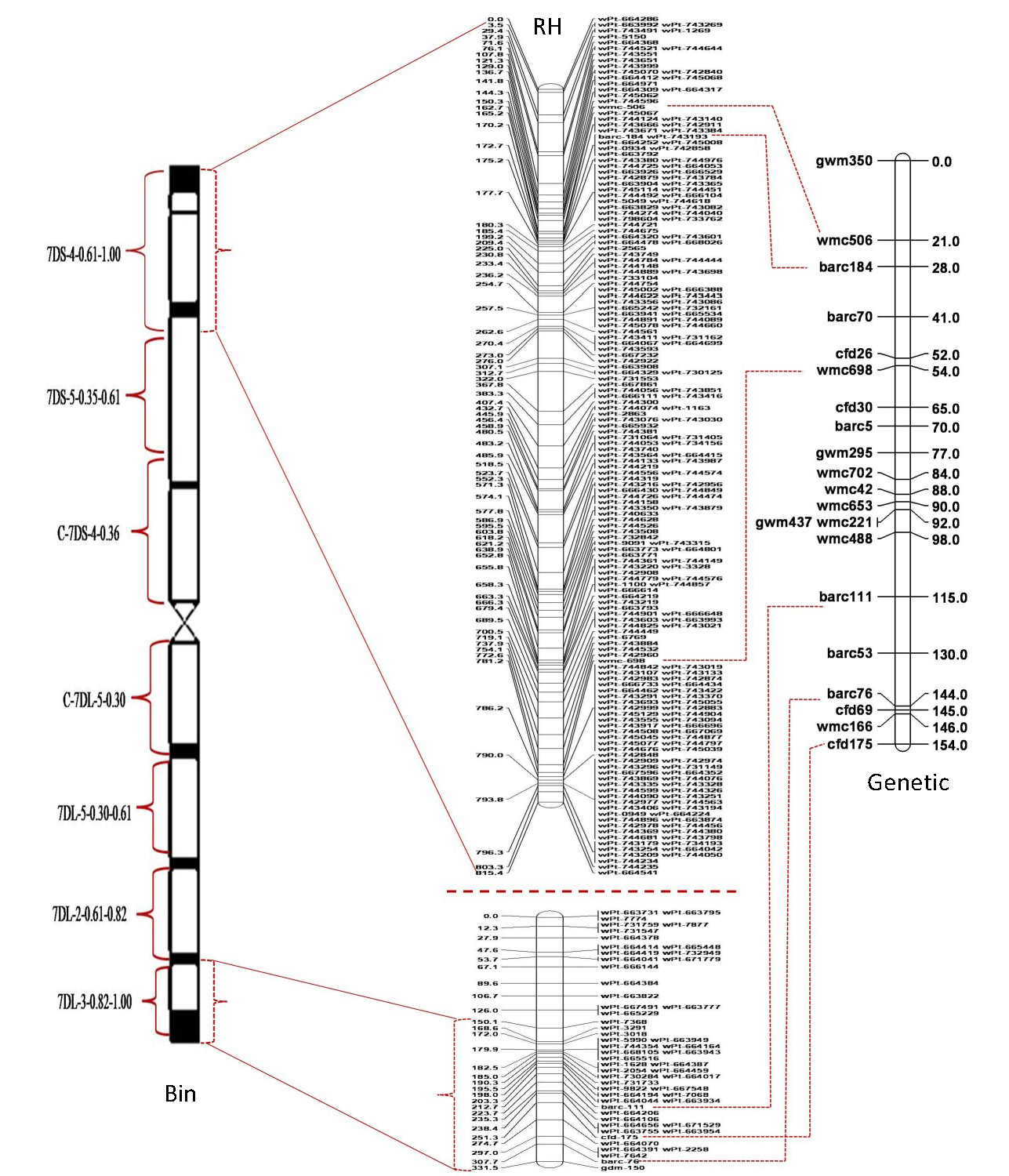

Supplement: Figure S7 — Radiation hybrid map of chromosome 7D. Map comparison of chromosome 7D as described in Figure S1. (TIF) [file pone.0048815.s007.tif]

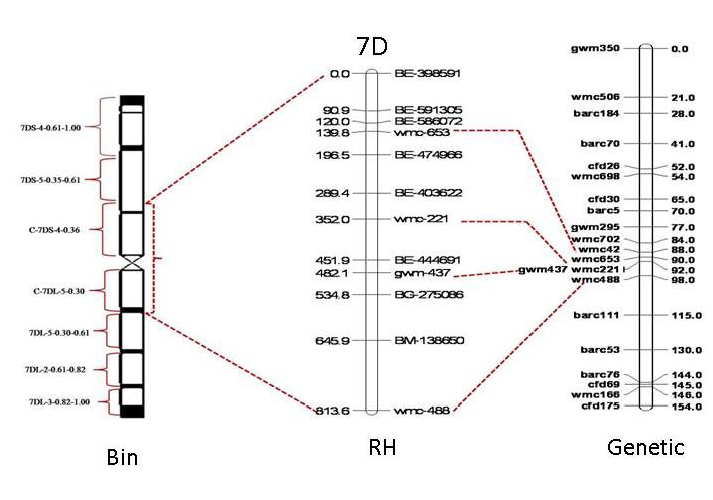

Supplement: Figure S8 — Radiation hybrid map of 7D pericentomeric region. Comparison of deletion bin map, RH map, and consensus genetic map of the pericentomeric region of wheat chromosome 7D. Eight ESTs and four SSRs were mapped to the low-recombination pericentromeric region of chromosome 7D. Dotted lines connect markers mapped to multiple maps. (TIF) [file pone.0048815.s008.tif]

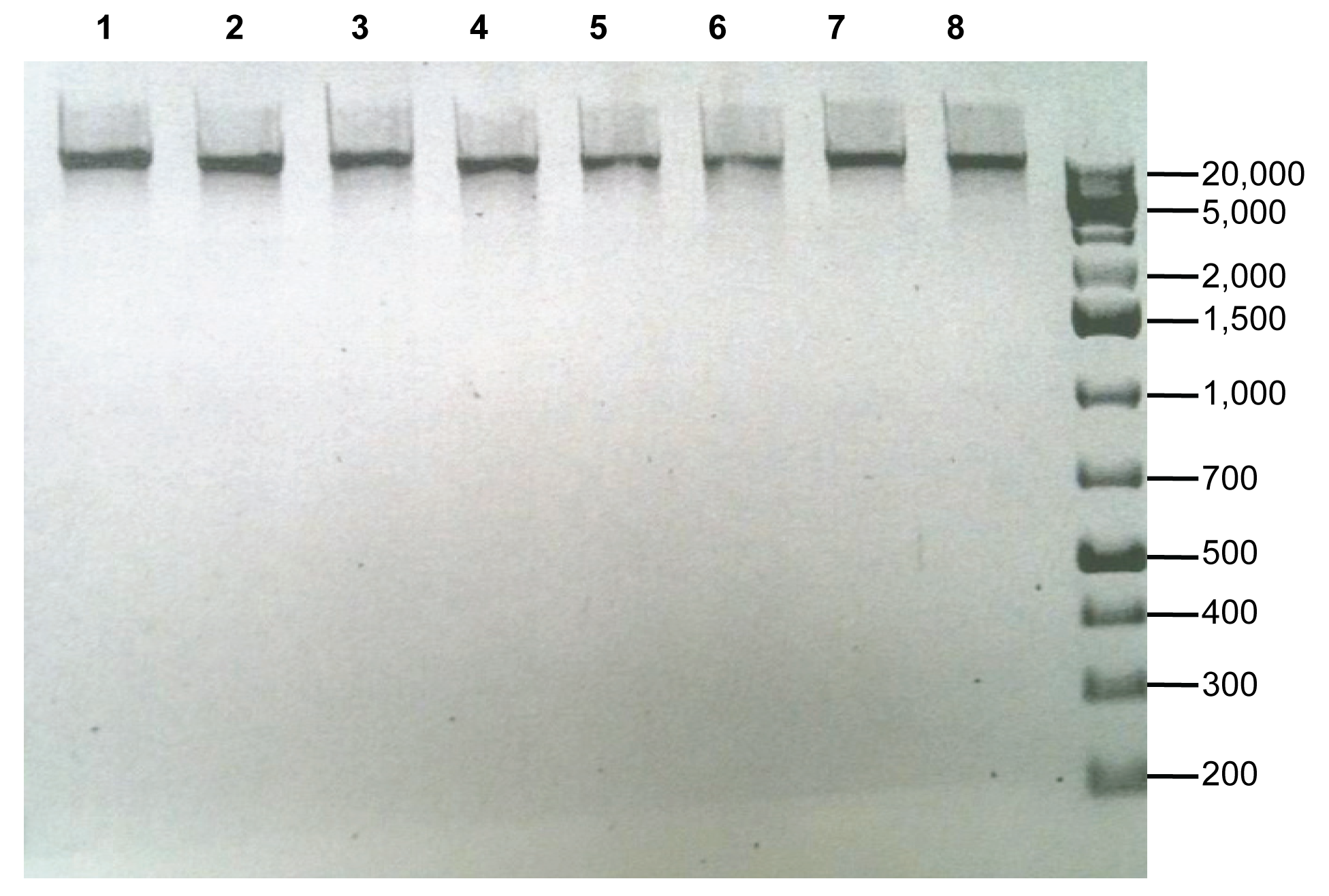

Supplement: Figure S9 — DNA recovered from endosperm is high molecular weight. Aliquots of endosperm DNA derived from 20-Gy-irradiated pollen were assayed by electrophoresis on a 0.8% agarose gel and visualized by staining with ethidium bromide. Lane 1 is unirradiated CS endosperm, lane 2 is unirradiated Altar endosperm and lanes 3 through 8 are 6 independent CS/Altar F1 endosperms. Numbers on right represent base pairs. (TIF) [file pone.0048815.s009.tif]
